# Developmental timing distinguishes pediatric and adult cancers through retention and rewiring mechanisms

**DOI:** 10.1038/s41467-026-75182-7

**Published:** 2026-07-01

**Authors:** Shayan Saniei, Elvin Wagenblast

**Affiliations:** 1 Computational Oncology, Memorial Sloan Kettering Cancer Center, New York, NY USA; 2 Tri-Institutional Program in Computational Biology and Medicine, Weill Cornell Medicine, New York, NY USA; 3 Department of Oncological Sciences, Icahn School of Medicine at Mount Sinai, New York, NY USA; 4 Department of Pediatrics, Division of Pediatric Hematology-Oncology, Icahn School of Medicine at Mount Sinai, New York, NY USA; 5 Department of Stem Cell Biology and Regenerative Medicine, Icahn School of Medicine at Mount Sinai, New York, NY USA; 6 Tisch Cancer Center, Icahn School of Medicine at Mount Sinai, New York, NY USA; 7 Mindich Child Health & Development Institute, Icahn School of Medicine at Mount Sinai, New York, NY USA; 8 Center for Advancement of Blood Cancer Therapies, Icahn School of Medicine at Mount Sinai, New York, NY USA

**Keywords:** Cancer epigenetics, Cancer stem cells, Cancer, Paediatric cancer

## Abstract

Oncogenic mutations can have fundamentally different consequences depending on the developmental state of the tissue in which they arise. We propose a unifying model in which early-life oncogenic events promote retention, stabilization of transient embryo-fetal transcriptional and epigenetic programs, whereas later-life mutations more often require rewiring, reactivating suppressed oncofetal states through stepwise transcriptional and chromatin remodeling. This temporal dimension helps explain why childhood and adult cancers can follow distinct lineage trajectories, behaviors, and therapeutic responses, even when initiated by the same genetic lesion. Incorporating developmental timing into cancer modeling and risk stratification may improve prognostic resolution and reveal stage-specific vulnerabilities.

## Abstract (web summary)

Oncogenic mutations have different effects depending on tissue developmental stage. Here, the authors propose that early-life events preserve embryo-fetal programs, while later-life mutations reactivate them through rewiring, which may have clinical implications.

## Introduction

Pediatric and adult tumors are distinguished by well-established molecular differences, including a markedly lower mutational burden in childhood and young adult cancers. Pediatric tumors are enriched for copy number alterations and structural variants^1^ rather than the accumulation of large numbers of point mutations, and fewer than half of recurrent pediatric alterations are represented in pan-adult mutational landscapes^2^. The tissue origins and cancer types most frequently observed across age groups are also distinct^3^. Adult cancers predominantly arise from epithelial compartments in the gastrointestinal tract, lung, breast, and reproductive organs, whereas pediatric cancers are disproportionately composed of brain tumors^4,5^, hematological malignancies^2^, and embryonal/endocrine cancers^6^. Additionally, many pediatric solid tumors originate from mesodermal, ectodermal, and endodermal tissues that are still developing during embryonic life, transient structures that are no longer present in adulthood^7^. Together, these contrasts argue that oncogenic selection operates on different cellular substrates across the lifespan, with distinct sets of transforming events becoming advantageous in different tissues and developmental windows.

**Fig. 1:**
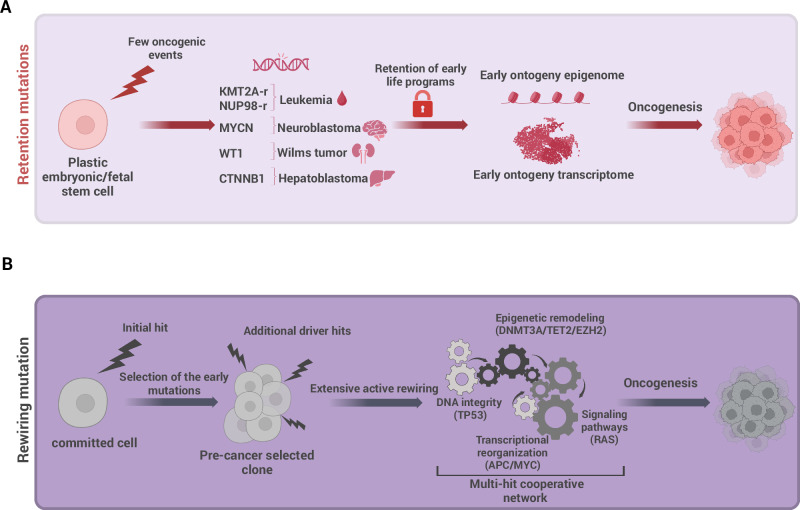
Retention versus rewiring mutations. **A** Early-life oncogenic lesions (such as KMT2A rearrangements, NUP98 rearrangements, MYCN, WT1, and CTNNB1 mutations) arising in plastic embryonic or fetal stem cells promote retention of early developmental programs by leveraging an already permissive epigenomic and transcriptional landscape. **B** In contrast, late-life oncogenic lesions (such as DNMT3A, TET2, EZH2, APC, MYC, RAS, and TP53 alterations) typically require multi-hit cooperative networks affecting epigenetic remodeling, DNA integrity, and signaling pathways to extensively rewire adult cells toward earlier developmental states, thereby promoting oncogenesis. (Created in BioRender. Wagenblast, E. (2026) https://biorender.com/ker6w2k).

**Fig. 2:**
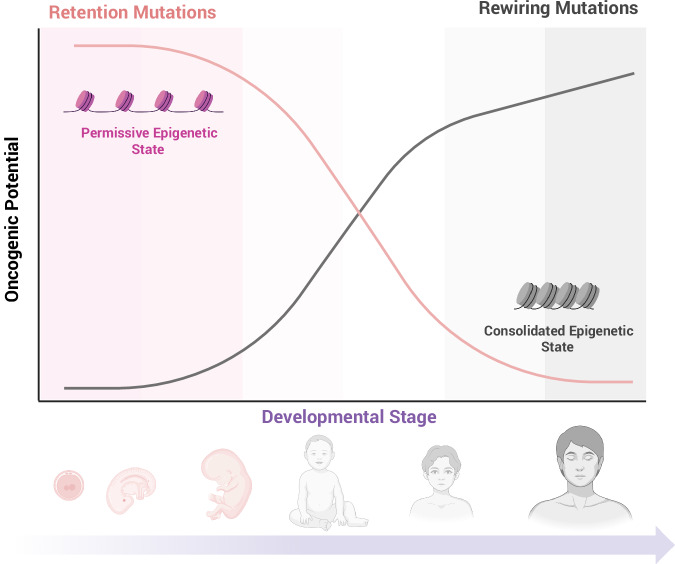
Retention-to-rewire spectrum across ontogeny. Retention and rewiring mutations can be conceptualized as a dynamic spectrum. In early life, when the epigenetic landscape is still broadly permissive, retention mutations carry higher oncogenic potential. In contrast, later in life, when the epigenetic landscape has consolidated, rewiring mutations confer a greater fitness advantage. (Created in BioRender. Wagenblast, E. (2026) https://biorender.com/q1wzr98).

A defining yet often underappreciated feature of pediatric cancers is that many initiating lesions arise during the most developmentally dynamic stages of human life^3^. Mounting evidence supports a fetal^8–13^, and in some cases embryonic^14,15^, origin for multiple malignancies, including leukemias, blastomas, and Wilms tumor. During development, cellular identity, chromatin accessibility, transcriptional circuitry, and niche dependence are rapidly remodeled. As a result, the same genetic lesion can confer a radically different selective advantage depending on when during development it occurs, even within the same lineage. This principle extends beyond pediatric cancers, as tumors across all ages can arise from stem or progenitor compartments, but these populations themselves traverse discrete maturation states with different proliferative capacity, metabolic wiring, differentiation potential, and epigenetic competence^16,17^. Here, we propose that developmental timing is a core determinant of oncogenic potency and downstream tumor biology. We recently established this principle experimentally in pediatric leukemia, where the same oncogenic lesion conferred different oncogenic potential and therapy responses depending on the developmental stage in which it arose^18^. Specifically, early-life oncogenic events may promote malignant evolution by retaining transient embryo–fetal transcriptional and epigenetic programs that are normally extinguished after birth (Fig. 1A). By stabilizing these permissive states, such lesions can sustain proliferation, alter differentiation trajectories, and enable long-term clonal persistence with comparatively few additional hits. Mutagenic processes resulting in early-life oncogenic events could also be development-dependent, where mutation-inducing pathways that generate these particular lesions are only active during narrow windows of development. In contrast, oncogenic events arising later in life more often promote reactivation of similar developmental and early-life programs that have been silenced in more lineage-restricted contexts. Although some adult genetic lesions such as IDH and EZH2 can drive an expeditious onco-fetal rewiring on their own via few hits^19,20^, other adult cancer-associated mutations must cooperate in a stepwise transcriptional reorganization and chromatin remodeling network to induce and promote adult cancers (Fig. 1B). This retention-to-reactivation spectrum (Fig. 2) provides a unifying explanation for why childhood and adult cancers often differ in mutational landscapes, lineage hierarchies, clinical behavior, and therapeutic response, even when driven by the same genetic lesion. As we will discuss in detail, this spectrum displays concordance with chronological age to an extent, but the epigenetic state of the substrate would be the ultimate predictor for selection of retention, rewiring, or a more nuanced combination of both classes of lesions. This concept also motivates incorporating developmental timing into cancer etiology, risk stratification, disease modeling, and precision treatment strategies.

More broadly, oncogenesis can be viewed as a constrained return toward earlier ontogenetic states. Early embryonic and fetal tissues naturally occupy highly plastic, growth-permissive states that overlap with canonical hallmarks of cancer, including proliferation, developmental signaling activity, and epigenetic permissiveness^21^. This perspective helps reconcile why pediatric cancers, while far less common than adult cancer overall, nonetheless arise at a notable frequency^22^, despite limited cumulative exposure to many exogenous mutagens. As discussed, pediatric cancer alterations could be caused by specific evolutionarily conserved and necessary developmental mutagens, such as rare off-target or aberrant hits. We additionally argue that in early life, the developmental starting state itself may reduce the number or complexity of events required to cross the threshold into malignant growth.

## Cancer stem cells recapitulate early ontogeny programs

Cancer stem cells (CSCs) were first prospectively identified in leukemia^23^ and later in several solid tumors, including breast^24^, colorectal^25^, and brain^26,27^ cancers, reframing tumors as hierarchically organized systems by self-renewing, therapy-resistant cell states^28^. This concept provides a mechanistic bridge to development: many CSC properties resemble programs deployed during early ontogeny, when stem and progenitor cells occupy highly plastic states governed by potent transcriptional and epigenetic circuitry. CSCs can occupy and revert to different states in a highly dynamic manner, displaying remarkable plasticity governed by their intrinsic characteristics, such as metabolomic and epigenetic plasticity, as well as by their unique interactions with diverse microenvironments and external pressures, including immune and therapeutic influences^29,30^. Although CSCs are not embryonic stem cells and lack true totipotency or pluripotency^31,32^, functional studies, including xenotransplantation assays, demonstrate that subsets of CSCs, such as leukemia stem cells, can display robust self-renewal and tumor reconstitution capacity that is comparable, in relevant respects, to the most potent tissue stem cells^15,33,34^. At the regulatory level, aggressive cancers frequently co-opt transcription factor networks associated with early developmental potency, including embryonic stem cell-like modules and core factors such as NANOG, OCT4, and SOX2^35^.

A major implication of this convergence is that developmental-like states can be achieved through different routes depending on timing. Key epigenetic regulators that control lineage specification in development, including Polycomb repressive complexes PRC1/PRC2, are repeatedly implicated in cancer stemness. Mutations or dysregulated activity of components such as EZH2 can promote oncogenesis and associate with stem-like phenotypes and adverse outcomes^36–38^. In general, alterations in epigenetic regulators account for nearly half of pan-cancer driver events, underscoring epigenetic dysregulation as a widespread and convergent oncogenic vulnerability. Their recurrent disruption may reflect the hijacking of developmental plasticity programs that enable tumor initiation and progression^39,40^. Likewise, MYC exemplifies a shared developmental-oncogenic axis. It supports self-renewal and growth program during development^41^ and is deregulated across a large fraction of human cancers, where it orchestrates proliferation, cell-state plasticity, immune evasion, and therapy persistence^42^. MYC also contributes to angiogenesis in both embryogenesis and tumor progression^43^, and MYC-centered transcriptional modules have been proposed as a key basis for similarities between embryonic and cancer-associated programs^44^. Together, these observations support the view that tumors repeatedly converge on early ontogeny circuitry, but whether this occurs through retention of already-active programs (early life) or reactivation via extensive remodeling (later life) may depend on developmental context.

Beyond cell-intrinsic circuitry, tumors and developing tissues share microenvironmental and physiological modules that can further reinforce oncofetal states. Developmentally conserved processes such as epithelial-mesenchymal transition (EMT) and extracellular matrix remodeling^45^ are frequently engaged in cancer progression^46,47^, while inflammatory signaling can promote wound-healing-like programs and foster fetal-like immunosuppressive niches, including expansion of fetal-like tumor-associated macrophage states^48–50^. In addition, therapy persistence programs have been linked to diapause-like adaptations in which MYC/Wnt activity is modulated to maintain survival under stress^51–53^. Thus, across ontogeny, both pediatric and adult cancers tend to hijack developmental programs either by retaining or recapitulating early-life programs. This provides a conceptual foundation for why the timing of oncogenic events can determine whether malignancy arises by stabilizing and retaining an already existing permissive developmental state or by actively rebuilding and rewiring cells towards such a primitive state if mutations occur later in life^23,54^.

## Early development retention mutations

It is well established that pediatric tumors carry a markedly reduced mutational burden, often more than tenfold lower than adult cancers^2^. Beyond overall mutational burden, pediatric cancers also differ from adult malignancies in the identity of driver lesions and in their patterns of acquisition and evolution. Early-life genomic lesions often arise as off-target consequences of essential developmental processes that govern maturation and establishment of healthy systems, such as activities of RAG1/2 DNA recombinase in shaping the lymphoid immune compartment and nuclease PGBD5 in the development of many solid and soft tissues^3^. Indeed, insightful evolutionary frameworks have argued that childhood mutations persist and have not undergone negative selection because the essential developmental processes that create them offset their oncogenic cost in the majority of developing children^3^. While some oncogenic pathways are shared across age, the specific genetic alterations and regulatory programs they engage can be distinct, emphasizing important developmental nuances rather than simple age-shifted parallels^1,2^. In a cohort of >1600 pediatric cancers, only 45% of the 142 identified pediatric driver genes were also recurrently observed in adult cancer datasets^2^. These age-associated driver profiles support the concept that oncogenic selection is developmentally contingent^3^. In early life, oncogenic lesions may gain a selective advantage by preserving and functionally locking in permissive embryonic or fetal transcriptional and epigenetic programs that are already active at the time of mutation. In such contexts, fewer cooperating events may be sufficient to achieve malignant transformation, resulting in a comparatively simpler mutational landscape. Clinically, this developmental dependency is evident in B-cell acute lymphoblastic leukemia (B-ALL). KMT2A rearrangements dominate infant B-ALL, ETV6-RUNX1 is enriched in childhood B-ALL, and BCR-ABL1, together with cooperating mutations, is a major driver in adult B-ALL. Notably, despite converging on a B-ALL phenotype, these subtypes exhibit distinct age-associated transcriptional programs^55^. A similar age dependence is observed in neuroblastoma, where MYCN alterations are more frequent in the youngest patients, whereas TERT and ATRX alterations occur at older median ages^56^.

Mutagenic processes can also be viewed through a developmental paradigm where cellular, enzymatic, and genomic machinery that facilitate mutagenesis of pediatric mutations are unique and constrained to early life^3^. RAG1/2 DNA recombinase, a mediator of somatic DNA rearrangements during early-life B and T cell development^57^, can also induce aberrant DNA alterations and produce mutations that lead to pediatric ALL or lymphomas^58^. Similarly, AID in lymphomas and PGBD5 in many childhood solid tumors, such as sarcomas and rhabdoid tumors, are typically active in early life as normal mediators of sequence-specific genomic alterations during healthy ontogeny, but can also cause aberrant off-target alterations in tumor suppressors and oncogenes, thereby initiating or promoting tumorigenesis in a highly developmentally constrained manner^59^. Consistent with this model, pediatric cancers are enriched for alterations in developmental regulators, transcription factors, and chromatin-associated genes that are highly active in embryonic and fetal life and later become restricted to immature progenitor states. Examples include recurrent alterations in RUNX1, WT1, MYCN, and TAL1 across pediatric malignancies^60^. Wilms tumor provides one of the clearest illustrations of an embryonal cancer origin. There, initiating lesions arise during kidney development and can generate premalignant clonal expansion within histologically normal tissue that later progresses to overt tumor^15^. Supporting a causal link between developmental timing and transcriptional state, deletion of WT1, a gene required for kidney development and for mesenchymal-to-epithelial transition (MET), prior to MET, yields defective kidneys with gene expression patterns that closely resemble WT1-mutated Wilms tumors^61,62^. Additionally, Wilms tumor exhibits a unique pattern of somatic mutagenesis^63^. Hepatoblastoma similarly exhibits strong developmental constraint. The majority of cases harbor CTNNB1 mutations with near-universal engagement of WNT signaling and enrichment of fetal and embryonic gene programs^64^. DNA methylation profiling has further shown that hepatoblastomas display fetal liver-like promoter methylation patterns and hypomethylated enhancers enriched for ASCL2 binding sites^65^, supporting the idea that transformation occurs in a developmentally immature hepatic progenitor population^66^. Given that most hepatoblastomas present in infants and young children (median age ~18 months)^67^, these observations are compatible with a prenatal or very early postnatal window of susceptibility in which Wnt/ASCL2-associated regulatory programs are active and can be reinforced by an initiation lesion. PAX3-FOXO1 oncogenic fusion protein promotes pediatric rhabdomyosarcoma by blocking differentiation toward committed muscle cells^68^.

Mutations in epigenetic regulators and chromatin remodelers are also prominent in pediatric cancers, with frequency varying by tumor types, and are particularly enriched in gliomas^69^, medulloblastomas^70^, and T-lineage acute lymphoblastic leukemia (T-ALL)^71^, where Compass/KMT2, PRC2, and SWI/SNF complexes are commonly impacted^72^. Conceptually, these lesions act as retention events by disrupting the epigenetic remodeling that normally accompanies maturation, thereby stabilizing the younger, more stem-like chromatin state that remains permissive for malignant evolution. For example, SMARCB1 (a SWI/SNF component) is frequently inactivated in teratoid/rhabdoid tumors, and its loss has been linked to impaired activation of differentiation programs and deregulated states that underpin aggressive disease^73,74^. Interestingly, rhabdoid tumors harbor genomic rearrangement signatures that are associated with PGBD5-mediated signal-sequence breakpoints, where inactivation of SMARCB1 itself can be the result of PGBD5’s genomic transposition activity^75^. PGBD5-mediated SMARCB1 loss in early life is a tangible and direct demonstration of the generation of a retention lesion by a developmental mutator. Another well-studied class of developmentally contingent epigenetic lesions involves the rearrangement of KMT2A (also known as MLL)^76^, which also captures how both genetic and epigenetic events converge on stabilizing early-life developmental states.

Rearrangements of KMT2A that fuse the gene to diverse partners occur across hematological malignancies, including B-ALL, acute myeloid leukemia (AML), and mixed-phenotype acute leukemia (MPAL)^76^. Humanized leukemia models generated by CRISPR/Cas9 have shown that oncogenic output depends not only on the immunophenotypic cell in which the rearrangement occurs, but also the developmental stage of that cell, directly implicating ontogeny as a determinant of transcriptional wiring and disease phenotype^77^. For instance, CRISPR/Cas9 induction of KMT2A-AFF1 (MLL-AF4), a hallmark lesion in infant B-ALL, in human fetal liver hematopoietic stem and progenitor (HSPCs) drives infant-like B-ALL in vivo and recapitulates fetal-associated gene expression programs observed in patients. In contrast, engineering KMT2A-AFF1 into postnatal cord blood HSPCs yields a disease that more closely resembles childhood B-ALL that lacks key fetal programs^77^. Single-cell multiome analyses comparing younger and older KMT2A-rearranged patients have revealed distinct blast cell states in younger patients marked by increased cellular plasticity and immunosuppressive features^78^. Extending this concept even earlier, single-cell omics phylogeny reconstruction studies suggest that, in some cases, KMT2A rearrangements may occur during embryogenesis, potentially before hematopoietic specification, consistent with an extremely early window of oncogenic competence^79^.

A closely analogous phenomenon is observed in NUP98-NSD1-driven pediatric AML. NUP98, a nuclear pore complex component, forms fusion oncogenes with a variety of partners, including homeobox (HOX) genes, histone demethylase KDM5A, and most commonly the histone methyltransferase NSD1^80–82^. Distinct NUP98 fusions show different ages of onset and transcriptional programs. For example, NUP98-KDM5A tends to present earlier in childhood and is associated with acute megakaryoblastic leukemia (AMKL)^80,83,84^. NUP98-NSD1, the most frequent NUP98 fusion, commonly co-occurs with cooperating lesions such as WT1 loss-of-function and FLT3 alterations, defining a high-risk AML subset with poor outcomes^85,86^. Importantly, controlled CRISPR/Cas9 engineering of NUP98-NSD1 into immunophenotypically similar human HSPCs across ontogeny, including fetal liver, postnatal cord blood, pediatric bone marrow, and adult bone marrow, revealed striking developmental specificity. Fetal HSPCs readily initiated AML in vivo, postnatal cells required cooperating lesions (such as WT1 loss) and generated less plastic leukemias with altered hierarchies, and pediatric and adult bone marrow HSPCs remained resistant to transformation even with cooperating events^18^. These findings support a time-dependent oncogenesis paradigm in which the same translocation, occurring in a similar stem and progenitor compartment, can be permissive, constrained, or ineffective depending on developmental context.

Mechanistically, hematopoietic stem cells (HSCs) offer a tractable system to understand how intrinsic maturation states might gate oncogenic competence. HSC properties change dynamically with age and developmental stage^16^. During definitive hematopoiesis, as HSCs emerge from hemogenic endothelium and expand in the fetal liver, they are highly proliferative and engage developmental inflammatory-niche signaling programs, including Notch-associated circuits^87–89^. As HSCs seed the bone marrow around birth and establish lifelong hematopoiesis, they transition towards a more quiescent, dormancy-prone state that protects against exhaustion^16^. This developmental shift occurs rapidly: in humans, telomere fluorescence measurements are consistent with a major transition within the first year of life^90^, while in mice, many of the most potent HSCs enter quiescence by ~3–4 weeks, accompanied by reduced self-renewal capacity^87,91^. Transcriptomic comparisons between young and aged HSCs further reveal systematic changes in self-renewal programs and lineage priming^92–95^, and epigenomic profiling demonstrates progressive remodeling of DNA methylation, chromatin accessibility, and histone marks that cumulatively silence developmental and self-renewal programs with age^96–98^. This ongoing transcriptional and epigenetic maturation provides a plausible biological basis for the pronounced ontogeny dependence observed in KMT2A- and NUP98-rearranged leukemias. Early HSCs, and potentially their embryonic precursors, may rely more heavily on regulatory networks controlled by KMT2A, NUP98, and their fusion partners, whereas this dependency diminishes as epigenetic aging closes developmental enhancers and restricts cellular competence. Consequently, the same initiating lesion can function as a potent retention event early in life but lose transformative capacity as development proceeds and epigenetic state evolves past early life’s permissiveness. While hematopoiesis offers especially clear experimental evidence, the same logic likely extends to pediatric solid tumors in which transient developmental programs define narrow windows of susceptibility.

## Late ontogeny embryo–fetal rewiring mutations

We postulate that as development progresses, early retention mutations lose the transformative potency they possess during early ontogeny and are no longer sufficient, on their own, to drive oncogenesis. At these later developmental stages, lesions that actively re-establish embryo–fetal transcriptional and epigenetic programs are more likely to be selectively favored. In this embryo–fetal rewiring model, transformation occurs in cells that have partially or fully exited their developmental competence window for early-life ‘retention’ lesions, cells whose chromatin has already been remodeled toward a more lineage-restricted state. Oncogenic events must therefore act against an epigenetically closed backdrop, reopening developmental loci that were progressively silenced, rebuilding regulatory circuitry, and in some contexts perturbing stable epigenetic memory established during maturation. Conceptually, these mutations do not merely pause differentiation; they reverse aspects of ontogenetic progression and promote de-differentiation towards fetal and embryonic-like states that the cell has already left behind. Such extensive reprogramming typically requires a stepwise, multi-hit sequence of hits impacting genome integrity, chromatin regulation, and signaling pathways to assemble a cooperative rewiring network. Consistent with this logic, rewiring-type alterations and their associated trajectories become increasingly prominent from later pediatric ontogeny into adolescence and young adulthood, when cellular commitment and epigenetic consolidation make active reactivation necessary. We acknowledge that not all adult tumors conform to this pattern of mutagenesis. Notable exceptions include chromoplexy-driven cancers^99^ and single-hit tumors such as Burkitt lymphoma^100^. Nevertheless, the mutational landscape of most adult tumors reflects interconnected, cooperative alterations rather than isolated events.

Insights from induced pluripotent stem cell (iPSC) biology provide a useful analogy: the adult somatic epigenome can be a barrier to reprogramming, and early epigenetic remodeling events, such as those mediated by TET2 and PARP1, often precede efficient acquisition of pluripotency^101^. A similar principle may apply to oncogenesis in mature tissues. Early lesions may first reshape the chromatin and transcriptional state, increasing plasticity and partially reverting aspects of cellular identity, thereby creating a permissive substrate on which additional driver mutations can be selected and integrated into a malignant program. It is worth emphasizing that, as noted in the previous section, alterations in epigenetic regulators are also among the main pan-cancer drivers of childhood cancers, and importantly, epigenetic modifications can precede oncogenic mutations and increase predisposition to certain pediatric cancers, such as Wilms tumor^102,103^, where epigenetic mutations and non-genetic modifications stabilize the already permissive epigenetic state. Clonal hematopoiesis (CH) exemplifies this staged evolutionary logic in a more adult context: initiating mutations confer a competitive advantage and enable long-term clonal expansion of HSPCs, which then serves as fertile ground for the acquisition of additional drivers that culminate in overt malignancy. The most common initiating mutations in CH occur in epigenetic regulators, particularly DNMT3A and TET2, highlighting how altered epigenetic regulation can create a durable premalignant state^104^. DNMT3A catalyzes DNA methylation at CpG sites, whereas TET2 promotes DNA demethylation through oxidation of 5-methylcytosine, together governing key aspects of hematopoietic epigenetic homeostasis^105,106^. DNMT3A- and TET2-mutant CH is frequently associated with broad DNA methylation changes and epigenetic dysregulation^107,108^; however, the precise mechanisms linking these methylation shifts to transcriptional output can be context dependent, particularly for DNMT3A, where methylation changes do not necessarily translate into uniform gene expression shifts^109^. Nonetheless, experimental models support the concept that DNMT3A loss can establish a premalignant expansion that cooperates with later lesions (such as RAS pathway activation) to enable leukemic transformation^110^. In parallel, TET2 loss can induce hypermethylation at enhancers and transcription factor binding sites involved in hematopoietic differentiation, creating a cancer-prone state. In AML, TET2 alterations frequently co-occur with additional driver events such as NPM1, ASXL1, and IDH mutations^111–113^. Notably, long-read single-cell transcriptomic analyses of CH have suggested that DNMT3A- and TET2-mutant HSCs can appear transcriptionally younger than co-resident wild-type HSCs within the same niche, consistent with the idea that early epigenetic lesions possibly blunt age-associated transcriptional drift (in a mechanism similar to ‘retention’ mutations) or ‘rewire’ the epigenetic programs towards a more developmentally permissive state, priming the mutant cells for further genomic alterations^95^. CH highlights the nuances of our proposed retention-to-rewiring spectrum, where both mechanisms could be complementary and at play.

A vivid example of mutation-driven oncofetal rewiring in solid tumors is colorectal cancer. Loss-of-function alterations in APC, a tumor suppressor and a central negative regulator of Wnt signaling, are among the most common initiating events in colorectal tumorigenesis^114–117^. APC loss typically produces premalignant lesions that subsequently progress to carcinoma. Modeling APC inactivation has shown that early lesions upregulate Wnt pathway targets and reactivate gene programs associated with fetal intestinal states^118^. Further work identified RXR as a gatekeeper of oncofetal reprogramming in this setting, which APC loss enables a fetal-like program sustained by AP-1 and YAP^52^. These oncofetal-rewired lesions can then acquire additional driver events (such as TP53 or KRAS mutations and/or 18q loss) to promote malignant progression^119^. In this framework, APC loss acts as an initiating rewiring event that reconfigures cellular states and creates the permissive ground on which later mutations yield sharper fitness gains.

Differences between MYCN and MYC provide another lens on the retention-rewiring dichotomy. Although both belong to the MYC family, they differ in developmental expression, regulatory control, and transcriptional kinetics^120,121^. MYCN plays a central role in neurogenesis and brain development by supporting the expansion of neural progenitor populations^122^. Accordingly, MYCN amplifications occur in up to 20% of neuroblastoma cases, a tumor that largely presents in early childhood, with a median age at diagnosis of ~17 months^123–125^. Neuroblastoma is thought to arise from neural crest-derived progenitors, and tumor cells often recapitulate fetal neuroblast transcriptional programs^126,127^. MYCN amplification is proposed to reinforce these existing developmental circuits, potentially through enhancer invasion, thereby enforcing a differentiation blockade and stabilizing a fetal-like state^127,128^. Focused studies have implicated several members of the SAGA transcriptional coactivator complex as unique dependencies of MYCN-driven neuroblastoma, further supporting the view that this tumor represents a dysregulated developmental state^129^. In this sense, MYCN can be viewed as a retention-like driver that fortifies programs already active in the susceptible developmental window.

By contrast, MYC is often described as a global transcriptional amplifier that regulates 10–15% of human genes and supports cell cycle progression, growth, metabolism, and survival^130,131^. MYC is most commonly activated by overexpression of an otherwise intact protein through translocations, amplifications, or other regulatory alterations^132^, and is implicated across diverse hematological and solid tumors^133–135^. Notably, MYCN can substitute for MYC during embryogenesis in some contexts, whereas the converse appears less feasible, consistent with partially specialized developmental roles^136,137^. In many tumor types, MYC activation alone is typically insufficient for full transformation and instead operates with cooperative mutational networks (such as KRAS, TP53, and other lesions) that collectively reshape genomic stability, signaling metabolism, and the tumor microenvironment^138–141^. An instructive exception is Burkitt Lymphoma, where MYC translocation can function as a principal driver in mature B-cells, consistent with the idea that in certain permissive cellular contexts, MYC activation can mainly amplify an existing lymphoid transcriptional program rather than fundamentally rewiring cell identity^100,132,142^. Overall, these examples underscore how developmental context and baseline regulatory state determine whether oncogenic events act primarily by stabilizing an existing oncofetal program or by actively reconstructing one through multi-hit rewiring.

## Timing shapes trajectory and therapeutic vulnerability

As discussed above, the timing of mutational events across ontogeny is a major determinant of oncogenic competence and, should transformation occur, of the biological properties of the resulting cancer. A key concept in discussions of fetal, pediatric, and adult cancers is how the microenvironment differs across ontogeny and, importantly, how the immune system potentially interacts with cancer cells differently at each developmental stage. Lineage priming, proliferation, and self-renewal of developing tissues, and of some adult stem cells such as HSPCs and colorectal stem cells, are tightly orchestrated by specific cytokines such as interleukins and interferons produced in niche^143–145^. Importantly, intracellular production of cytokines in key microenvironmental compartments such as monocytes differs between fetal and adult life^146^. Thus, developmentally influenced cytokine-mediated crosstalk between cancer and its niche could differ depending on when in ontogeny transformation occurs, which could substantially reshape the tumor’s initiation, progression, and therapy response. It is important to emphasize that the immune system undergoes active development from fetal life through the postnatal period, and the fetal immune system is fundamentally different from a fully matured adult immune system. Recent work has shown that human T cell development is ‘layered’, where early-life T lymphocytes arising from fetal HSPCs are heavily enriched for regulatory T cells relative to other cytotoxic T lymphocytes, which typically arise in greater numbers after birth^147,148^. This layered immune development promotes an immune-tolerant environment in fetal life, when the fetus is in constant interaction with maternal antigens. Thus, tumors arising before birth are under the surveillance of a tolerogenic immune system, potentially facing lower immune pressure with minimal immunoediting of tumors. Specific immune therapies such as CAR-T therapy for CD19+ hematological malignancies have shown remarkable success in pediatric patients^149^; however, other immune therapies that are currently in the clinic for the treatment of adult cancers might not be as effective in pediatric cancers. Immunogenic cancer-specific neoantigens are less common in pediatric cancers due to their lower tumor mutational burden, and expanded CD8 T cell clones are rare in children^150^. Early-life lymphocytes also generate more muted type I and type II interferon responses and are unable to respond to certain antigenic stimuli^151^. Given these limitations, the development of effective and safe immunotherapies for children remains an active and thriving area of research. We therefore argue that time-dependent oncogenesis does not merely shape disease initiation; it also influences evolutionary trajectory, clinical behavior, and therapeutic vulnerabilities. In this framework, developmental context helps explain why cancers with overlapping genetic lesions can follow divergent routes to malignancy and respond differently to the same therapies across age groups

**Fig. 3:**
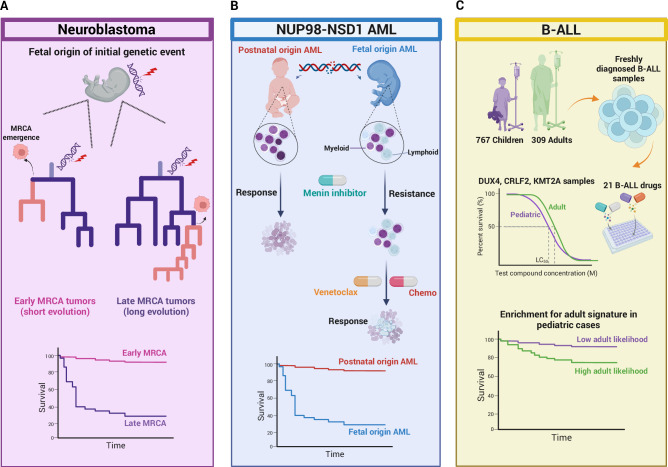
Developmental timing as a determinant of cancer trajectory and therapeutic vulnerability. **A** In neuroblastoma, although initiating genetic alterations arise during fetal life, two evolutionary dynamics appear to be at play. Prolonged prenatal evolution and a later emergence of the tumor’s most recent common ancestor (MRCA) are associated with more aggressive clinical behavior and poorer survival **B** In NUP98-NSD1 AML, fetal-origin disease exhibits greater lineage plasticity and aggressiveness and is resistant to menin inhibition, whereas postnatal AML remains sensitive to menin-targeted therapy. In contrast, combinations of venetoclax and chemotherapy may be more effective in fetal-origin AML, which is associated with inferior clinical outcomes compared with postnatal-origin disease, even when the initiating lesion is shared. **C** In B-ALL, freshly diagnosed samples from a large cohort of pediatric and adult patients displayed differential ex vivo responses to drugs commonly used in the clinic. This was particularly evident in DUX4-, KMT2A-, and CRLF2-rearranged samples, where adult cases harboring the same alterations exhibited higher LC50 values. Moreover, pediatric cases with transcriptional enrichment for adult-like disease showed increased treatment resistance and poorer prognosis. (Created in BioRender. Wagenblast, E. (2026) https://biorender.com/ohj64wr).

Neuroblastoma illustrates how the duration and timing of tumor emergence within a defined ‘retention’ window during fetal life can be clinically determinative. As one of the most common solid tumors of infancy and early childhood, neuroblastoma is increasingly recognized as having a prenatal origin, yet it displays striking heterogeneity in prognosis and clinical course^152^. Recent work using somatic single-nucleotide variants (SSNV) as a molecular clock to infer the timing of key evolutionary steps showed that major pathogenic alterations can occur as early as the first trimester (Fig. 3A). However, the inferred timing of the most recent common ancestor (MRCA) cell exerts outsized prognostic significance^153^. Across tumors, two evolutionary dynamics were observed: one in which early chromosomal gains coincide with early MRCA emergence (a shorter evolutionary course), and another in which early genetic alterations occur prenatally but MRCA emergence is delayed (a longer evolutionary course), even though the nature of early genomic events is broadly similar across the two classes^153^. Consistent with this model, later-MRCA tumors exhibited worse outcomes with shorter survival, and this effect persisted even when stratifying patients by MYCN amplification status. This differential clinical behavior might be related to the gain of telomere maintenance mechanisms during prolonged evolution of late MRCA tumors that underpins a more aggressive tumor. Notably, age at diagnosis alone was not strongly predictive, suggesting that clinically meaningful evolutionary differences can be encoded within prenatal developmental time, within a relatively narrow window, rather than simply reflecting postnatal duration of disease.

A conceptually related, but mechanistically distinct paradigm emerges in NUP98-NSD1-driven pediatric AML. CRISPR/Cas9 engineering of NUP98-NSD1 into immunophenotypically similar human HSPCs across early-to-late ontogeny demonstrated that induction of the same fusion oncogene into cells at progressively later developmental stages produces monotonically decreasing leukemogenic potential^18^. Importantly, fetal- versus postnatal-origin leukemias generated in vivo exhibited distinct therapeutic responses and prognostic features (Fig. 3B). Fetal-origin AML showed relative resistance to small-molecule menin inhibitors, which have recently gained regulatory approval for KMT2A-rearranged and NPM1-mutant AML, and have been proposed as candidate therapy for NUP98-rearranged AML^154,155^. In contrast, postnatal NUP98-NSD1 AML, modeled by engineering the fusion into cord blood HSPCs, showed robust sensitivity to menin inhibition. Moreover, among patients with NUP98-NSD1 AML, enrichment of an oncofetal transcriptional program (defined by comparison of fetal- and cord blood-origin leukemia stem cells) correlated with inferior survival, supporting clinical relevance of developmental state beyond the presence of the fusion itself. In this context, fetal-origin leukemia stem cells exhibited higher oxidative phosphorylation and increased dependence on BCL2-associated survival circuitry, providing a rational for venetoclax-based combination therapy^18^. Indeed, venetoclax plus chemotherapy produced near-eradication of disease in CRISPR/Cas9-based in vivo models and PDXs, whereas postnatal-origin AML displayed a comparatively attenuated response^18^. Collectively, these data support the premise that developmental origin can partition NUP98-NSD1 AML into biologically distinct disease states with different therapeutic liabilities, motivating developmental-aware stratification to reduce relapse and residual disease.

Specific subtypes of B-ALL, a malignancy with pronounced age-associated biology^55^, display a stark differential therapy response in pediatric versus adult cases to the same agents. In a large study evaluating ex vivo drug sensitivity of leukemia samples from 767 children and 309 adults with newly diagnosed B-ALL against 21 agents spanning six therapeutic classes, pediatric and adult samples showed significantly different LC50 values for multiple drugs (Fig. 3C)^156^. For mercaptopurine, a widely used antimetabolite in ALL therapy, adult samples were more resistant, whereas pediatric samples remained more sensitive, and this difference was observed largely independent of genetic subtype^157^. Notably, within CRLF2-, DUX4-, and KMT2A-rearranged subgroups, adult cases exhibited higher drug resistance than pediatric cases harboring the same lesions^156^. Unsupervised clustering further indicated that samples with similar mutational landscapes segregated predominantly by age-linked transcriptional state and drug response rather than by lesion alone^156^. Importantly, a subset of pediatric cases clustered with adult samples despite chronological age and displayed correspondingly higher drug resistance^156^. Enrichment of an adult-like transcriptional signature in pediatric cases was associated with poorer survival and treatment resistance^156^, suggesting that developmental epigenetic/transcriptional state can override chronological age and better capture clinically relevant biology.

Together, these examples, although methodologically not equivalent, broadly provide convergent evidence that developmental timing reshapes cancer trajectory and constitutes a clinically actionable dimension of prognosis and therapeutic vulnerability. At the same time, the direction and mechanistic basis of time dependence can differ across diseases. In neuroblastoma, prolonged prenatal evolution and later MRCA emergence are linked to more aggressive behavior^153^. In NUP98-NSD1 AML, fetal-origin disease is more therapy resistant and associated with inferior outcome relative to postnatal-origin disease, even when the initiating lesion is shared^18^. In B-ALL, cases carrying similar genetic lesions can exhibit markedly different drug sensitivities depending on their developmental transcriptional programs, and a subset of pediatric cases phenocopy adult-like resistance despite early age at presentation^156^. Collectively, these observations motivate a shift from viewing oncogenic lesions as context-free events to treating them as temporally contextual perturbations whose consequences and therapeutic liabilities are specified by developmental state.

## Future directions

In this perspective, we propose a paradigm in which oncogenesis and development are dynamically intertwined, continuously shaping and influencing one another. Despite limited cumulative exposure to exogenous mutagens, infants and young children develop cancers at notable frequencies, and a subset exhibits striking aggressiveness. This apparent paradox may reflect the deep biological parallels between early development and tumorigenesis. During early ontogeny, key features that later define malignant fitness, including rapid proliferative capacity and cellular plasticity, are physiologically present. Specifically, the intrinsically dynamic and flexible chromatin architecture of developing cells could render them particularly susceptible to broad oncogenic perturbations. Consequently, relatively few genetic alterations, resulting from essential developmental processes such as aberrant mutagenesis of early-life constrained DNA endonucleases or possible mutagens that cross the maternal-fetal boundary, may be sufficient to breach the boundary between normal development and malignant growth by retaining embryo–fetal programs. Although not a monotonic relationship, generally as development proceeds and epigenetic consolidation increases, the threshold for oncogenesis correspondingly rises: transformation increasingly requires a more complex and ordered accumulation of lesions that can reactivate or rewire developmentally silenced programs in more committed cells. Building on examples highlighted here, we advocate incorporating developmental origin into diagnostic and risk-stratification frameworks, particularly for biologically plastic borderline or early pediatric malignancies, in which developmental state may encode prognosis and therapy response.

**Fig. 4:**
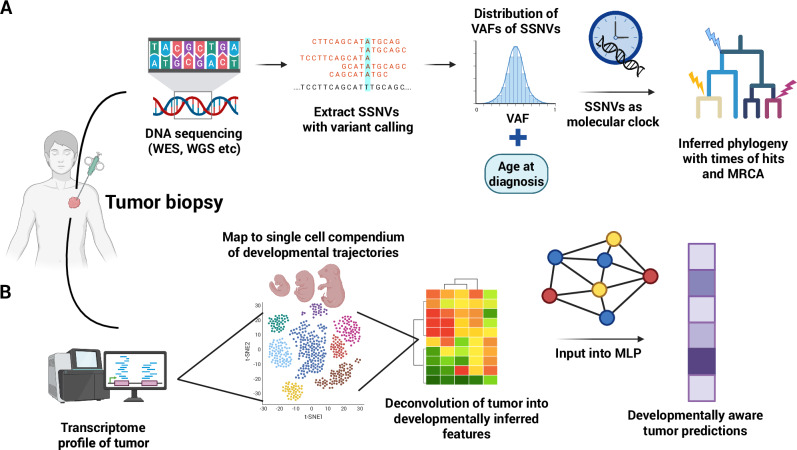
Development-aware cancer diagnosis in the clinic. **A** Somatic single-nucleotide variants (SSNVs) identified through genome sequencing can be used as molecular clocks, where the variant allele frequencies (VAFs) of SSNVs, together with the age at diagnosis, can inform phylogenetic reconstruction of tumors and enable inference of the timing of oncogenic events and the temporal emergence of the most recent common ancestor (MRCA) of the tumor. **B** Transcriptomic data from tumors can be mapped onto single-cell atlases of mammalian organogenesis to deconvolute tumor programs into developmental trajectories. These trajectories can then be used as inputs for a multilayer perceptron (MLP) classifier to predict tumor types and origins in a developmentally informed manner. (Created in BioRender. Wagenblast, E. (2026) https://biorender.com/f3rzueb).

The widespread availability of tumor genome and transcriptome data now makes it increasingly feasible to infer developmental timing in patients rather than treating it as an abstract concept. One practical strategy is to integrate variant allele frequencies (VAFs) with SSNVs as a molecular clock to estimate when key oncogenic events occurred and to approximate the timing of tumor-initiating clonal expansion relative to age at diagnosis (Fig. 4A)^153^. Inference frameworks grounded in population genetics can leverage time-dependent changes in VAF distribution to estimate the onset of clonal expansion leading to the clinically sampled tumor and the acquisition order and timing of critical alterations that precede or coincide with malignant transformation^153^. As shown in neuroblastoma, such approaches can reveal clinically meaningful timing differences even within prenatal development. A near-term priority is to extend and validate these models across larger, tumor-specific cohorts, especially in pediatric cancers where narrow developmental windows may confer outsized biological effects, and to integrate timing estimates with clinical endpoints to define development-aware prognostic groups that can be prospectively tested in clinical trials.

Artificial intelligence and machine learning offer complementary routes to operationalize a development-aware view of cancer by mapping tumors onto reference developmental programs. Developmental multi-layer perceptron (D-MLP), for example, first projects bulk tumor transcriptomes onto a compendium of developmental trajectories derived from >1 million single-cells samples across mammalian organogenesis, and then uses these developmentally informed features to classify tumor types that reflect specific developmental programs and demonstrates strong predictive performance (Fig. 4B)^158^. Beyond tumor typing, such developmental embeddings can be applied to cancers of unknown primary (CUP) to stratify cases by inferred developmental origin, thereby offering a conceptually grounded complement to purely tissue-of-origin classifiers. Parallel, smaller-scale machine learning models trained within specific disease contexts, including AML^159^ and brain cancers^160^, further support the feasibility of development-aware classification and risk prediction. A key next step will be harmonizing these computational outputs with mechanistic biology, linking inferred developmental programs to actionable state vulnerabilities, such as metabolic dependencies, chromatin regulators, and lineage plasticity that can be therapeutically targeted. It is of utmost importance that such predictive models are trained on diverse and demographically representative datasets that capture important variations and nuances, which would render such AI models generalizable to newly diagnosed patients in the clinic.

In conclusion, this perspective supports a developmentally driven framework of oncogenesis in which the selective advantage conferred by oncogenic lesions varies across the lifespan, transitioning through distinct competence states. While cancers arising in infancy and early childhood may appear counterintuitive, the overlap between early ontogeny and core malignant traits may render the earliest stages of life uniquely susceptible to transformation, often through retention of embryo–fetal programs, whereas later cancers more frequently require stepwise reactivation and rewiring. We propose that integrating developmental origin and ontogenetic context into clinical practice has the potential to refine prognosis, better predict therapy response, and guide the design of development-aware treatment strategies aimed at improving durability of response across pediatric and adult disease.
